# Expression and clinical significance of serum cystatin C in patients with hypertension and coronary heart disease

**DOI:** 10.1097/MD.0000000000020029

**Published:** 2020-05-29

**Authors:** Miaohui Zhao, Qingqing Che, Yandan Zhang, Xingjun Qian, Tong Huang

**Affiliations:** a Ningbo Medical Center Lihuili Hospital; b Cixi People's Hospital; c NingBo First Hospital, Ningbo, Zhejiang, China.

**Keywords:** clinical parameters, coronary heart disease, cystatin-C, hypertension

## Abstract

The aim of this study is to explore the potential association between cystatin C (Cys-c) and coronary heart disease (CHD) in hypertensive patients.

In this study, circulating levels of Cys-c in 62 essential hypertension (EH) patients, 147 hypertension with coronary heart disease (EH + CHD) patients, and 60 healthy volunteers were investigated using immunoturbidimetry. Then, we analyzed the correlations between Cys-C and other clinical parameters.

Serum Cys-C level was significantly higher in the EH and EH + CHD groups than in the control group, and higher in the EH + CHD group than in the EH group. Receiver operating characteristic curve (ROC) analysis showed that the diagnostic value of Cys-C for patients with hypertension combined CHD was 0.871(95% CI: 0.818–0.913). Serum Cys-C level was significantly higher in the double-vessel disease group and multi-vessel disease group than in the single-vessel disease group, and higher in the multi-vessel disease group than in the double-vessel disease group. Urinary albumin and CRP correlated positively with Cys-C, and HDL correlated negatively with Cys-C. Cys-C was an independent risk factor for CHD in hypertensive patients.

Our results suggested that circulating Cys-C levels was up-regulated in patients with hypertension and CHD, and had correlation with the severity of coronary artery disease. As one of the important risk factors for CHD, Cys-C can predict the occurrence of CHD in patients with hypertension.

## 1 Introduction

Coronary heart disease (CHD) is recognized as one of the most common cardiovascular diseases that treated human health.^[1]^ Although the incidence of CHD in developing countries is lower than in western developed countries, its incidence is increasing year by year.^[2]^ CHD is mainly caused by coronary atherosclerosis, but the specific pathogenesis remains unclear. Essential hypertension (EH) has been identified as an independent risk factor for CHD.^[3]^ Hypertensive combined with CHD can significantly reduce the patient's quality of life and bring a heavy burden to the family and society.^[4]^ Therefore, effective prevention and early detection of patients with hypertension and CHD are urgent.

Cystatin-C (Cys-C) is a non-glycosylated protein with a relatively low molecular mass of about 13.3 kDa. Cys-C is ubiquitous in the body fluids and tissue cells of the body. Its rate of production is extremely constant and is not affected by factors such as age, gender, and bilirubin.^[5]^ The only metabolic pathway of Cys-C is the kidney, where can be filtered by the glomerulus and completely reabsorbed in the proximal convoluted tubules and completely catabolized. Therefore, Cys-C is considered to be a sensitive indicator for the evaluation of renal impairment.^[6]^ Recent studies have shown that Cys-C is involved in many pathophysiological processes in the cardiovascular system.^[7]^ Epidemiological studies have shown that Cys-C is superior to creatinine or the glomerular filtration rate (GFR) based on creatinine-derived equations in predicting cardiovascular morbidity and mortality, particularly in elderly patients and in general populations without known chronic kidney disease.^[8]^ Thus the relationship between Cys-C and cardiovascular risk may be independent of renal function. Cys-C may be a marker of cardiac involvement in hypertensive patients,^[9]^ and it is also associated with the first ischemic cardiovascular event in patients without CHD, and this correlation reflects the non-renal aspect of the biological effects of Cys-C.^[10]^ Koening et al^[11]^ found that Cys-C is associated with risk of cardiovascular events and is associated with poor clinical outcomes in patients with CHD, revealing that Cys-C is independent of renal function and CHD.

At present, there are few reports on the correlation between serum Cys-C and hypertensive patients with CHD. We aimed to investigate the relationship between serum Cys-C levels and CHD in patients with hypertension in order to provide a reference for the clinical diagnosis and prognosis of patients with hypertension and CHD.

## 2 Materials and Methods

### 2.1 Study population

The clinical data of 209 patients with EH who were hospitalized at Ningbo Medical Center Lihuili Hospital from June 2016 to June 2018 were retrospectively analyzed. Depending on the presence of CHD, 62 patients were included into EH group and 147 patients were included into hypertension and CHD (EH + CHD) group. The diagnostic criteria for hypertension were based on the criteria for hypertension in the Chinese Hypertension Prevention and Control Guide (2010 revision), systolic blood pressure (SBP) ≥140 mm Hg and/or diastolic blood pressure (DBP) ≥90 mm Hg. Diagnosis of CHD was made according to the results of coronary angiography, at least one coronary artery stenosis ≥50% being the threshold for the diagnosis of CHD. Patients with infections, autoimmune disease, metabolic disease, and severe chronic diseases (such as thyroid disease, chronic kidney disease, secondary hypertension), GFR <60 mL/min·1.73 m^2^) and alcoholics were excluded from the study. We recruited 60 gender, body mass index (BMI), and age-matched healthy volunteer from the hospital's physical examination center from June 2016 to June 2018. We gathered all the participants’ basic health information by performing questionnaires and physical examinations to ensure that healthy volunteers had normal ECG and no history of cardiovascular disease such as hypertension or CHD. The research process and research methods were approved by relevant institutions of Ningbo Medical Center Lihuili Hospital. Informed consents were obtained from all volunteers.

### 2.2 Determination of clinical and biochemical indicators

General data (age, gender, height, weight, SBP, and DBP) of the subjects were collected and the BMI was calculated. Venous blood and urine were obtained after fasting overnight on the second morning after admission. AU5800 automatic biochemical analyzer and supporting reagents (Beckman Coulter, Miami, FL) were used to detect triglyceride (TG), low density lipoprotein (LDL), high density lipoprotein (HDL), total cholesterol (TC), and biochemical indicators such as apolipoprotein A1 (apoA1), apolipoprotein B (apoB), serum creatinine (sCr), urinary albumin, blood uric acid (sCr), and fasting blood glucose (FBG). The GFR was calculated as follows: GFR (mL/[min•1.73m^2^]) = ([140-age] × body weight [kg] × 1.228)/sCr (μmol/L). For woman, multiplication by 0.85 was used for correction. Serum C-reactive protein (CRP) and interleukin-6 (IL-6) levels were measured using an enzyme-linked immunosorbent assay (ELISA) kit (Shanghai Ji Ning Industrial Co., Ltd, China).

### 2.3 Coronary angiography

In hypertensive patients with typical symptoms of angina pectoris and typical ischemic ECG changes (new or transient ST-T depression ≥0.1 mV or T wave inversion ≥0.1 mV), coronary angiography was performed by a skilled cardiologist. Finally, patients with EH and CHD were grouped according to the results of the angiography. At least one coronary artery stenosis ≥50% led to the diagnostic of CHD. If there was a stenosis in the left anterior descending (LAD), left circumflex (LCX), or right coronary artery (RCA), it was classified as single-vessel disease group. If there were 2 lesions, it was classified as double-vessel disease group. For left main (LM) lesions, regardless of LAD or LCX lesions, they were also classified as double-vessel disease group. The multi-vessel disease group included all those patients with 3 or more lesions.

### 2.4 Statistical analysis

Data analyses were carried out with SPSS17.0 for Windows (IBM, Chicago, IL). Values were expressed as mean ± standard deviation (SD) for normally distributed variables. Variables with skewed distributions such as CRP, IL-6, the log-transformed were used in the analysis. After the homogeneity of variance test, comparisons of parameters were analyzed by Student unpaired *t* test, one-way analysis of variance or Chi-square tests when appropriate. Pearson or Spearman correlation coefficient test was used for the univariate relation between variables. The receiver operating characteristic curve (ROC) was used to analyze the diagnostic value of Cys-C level in patients with EH and CHD. Multiple linear regression was applied to analyze the relationship between the variables and the confidence interval (CI) was 95%. Results were considered statistically significant at *P* < .05.

## 3 Results

### 3.1 Comparison of the basic data of EH group, EH + CHD group and control group

**Figure 1:**
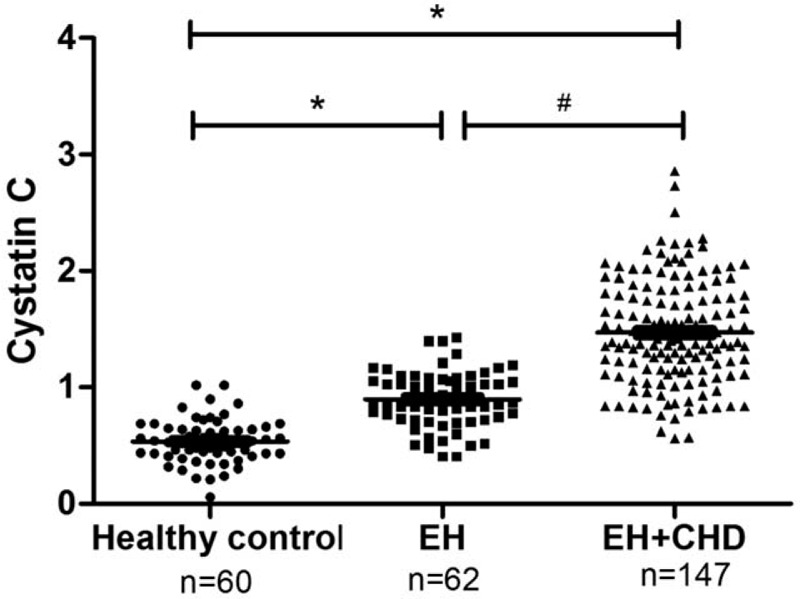
Levels of Cys-C from healthy control group, essential hypertension (EH) group and hypertensive patients with coronary heart disease (EH + CHD) group. EH and EH + CHD patients had a significantly elevated Cys-C levels when compared with healthy controls. Levels of Cys-C was higher in the EH + CHD group than in the EH group. ^∗^*P* < .05 compared with healthy control; ^#^*P* < .05 compared with EH group.

As shown in Table 1, patients in the EH and EH + CHD groups were significantly older than those in the control group (*P* < .05). BMI, SBP, DBP, apoB, urinary albumin, CRP, and IL-6 were significantly higher in the EH and EH + CHD groups than in the control group (*P* < .05), and higher in the EH + CHD group than in the EH group (*P* < .05). Compared with the control group and EH group, LDL was significantly increased in EH + CHD group, while HDL was significantly decreased (*P* < .05). The levels of GFR in the EH group and the EH + CHD group were significantly lower than the healthy control (*P* < .05), but there was no significant difference between the EH group and the EH + CHD group. Serum Cys-C level was significantly higher in the EH and EH + CHD group than in the control group, and higher in the EH + CHD group than in the EH group (Table 1 and Fig. 1). Other characteristics did not differ significantly among groups.

**Table 1:**
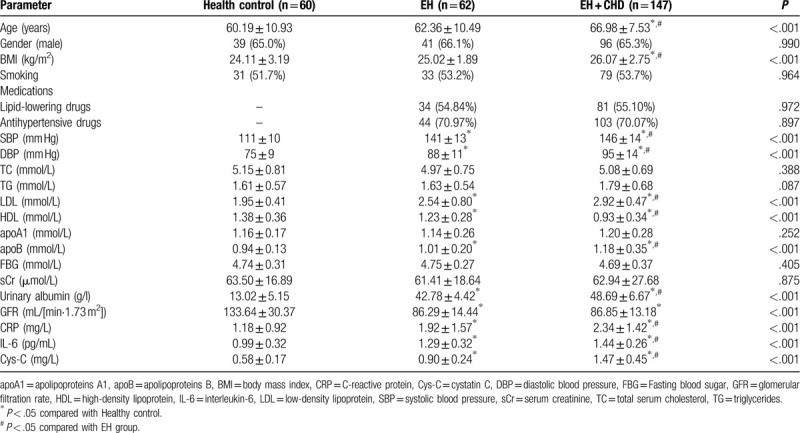
Comparison of clinical basic data at admission.

### 3.2 The predictive value of Cys-C in patients with hypertension complicated with CHD

**Figure 2:**
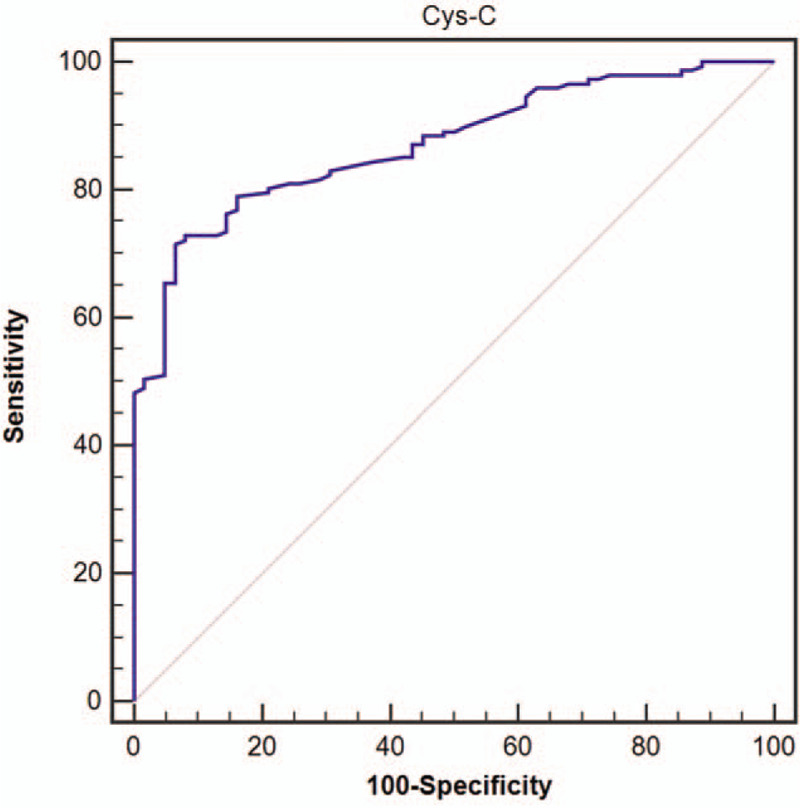
ROC curve analysis of the diagnostic value of Cys-C for hypertensive patients with coronary heart disease.

In view of the differences in Cys-C levels between EH and EH + CHD patients, we further used ROC to analyze the diagnostic value of Cys-C for hypertension with CHD. As shown in Figure 2, the area under the ROC curve for Cys-C diagnosis of EH + CHD was 0.871 (95% CI: 0.818–0.913, *P* < .01), sensitivity was 71.43% and specificity was 93.55%.

### 3.3 Relationship between Cys-C and the number of coronary artery lesions in hypertensive patients with CHD

**Figure 3:**
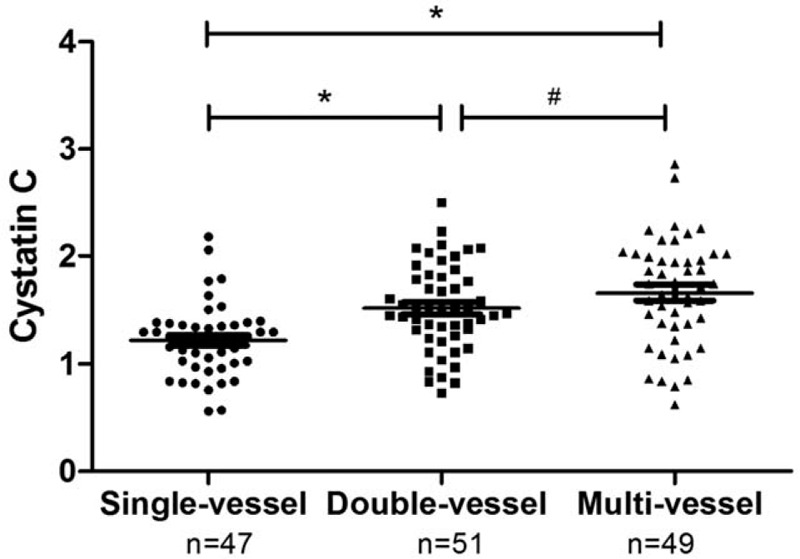
Levels of Cys-C from single-vessel disease group, double-vessel disease group and multi-vessel disease group. Double-vessel and multi-vessel disease patients had a significantly elevated Cys-C levels when compared with single-vessel disease patients. Levels of Cys-C were higher in the multi-vessel disease group than in the double-vessel disease group. ^∗^*P* < .05 compared with single-vessel disease control; ^#^*P* < .05 compared with double-vessel disease group.

We analyzed the relationship between Cys-C and other clinical parameters and the severity of hypertension in patients with CHD. As shown in Table 2, compared with the single-vessel disease group, BMI, DBP, LDL, urinary albumin in the double-vessel disease group and multi-vessel disease group were significantly increased, while HDL was significantly decreased (*P* < .05). Serum Cys-C level was significantly higher in the double-vessel disease group and multi-vessel disease group than in the single-vessel disease group, and higher in the multi-vessel disease group than in the double-vessel disease group (*P* < .05, Table 2 and Fig. 3).

**Table 2:**
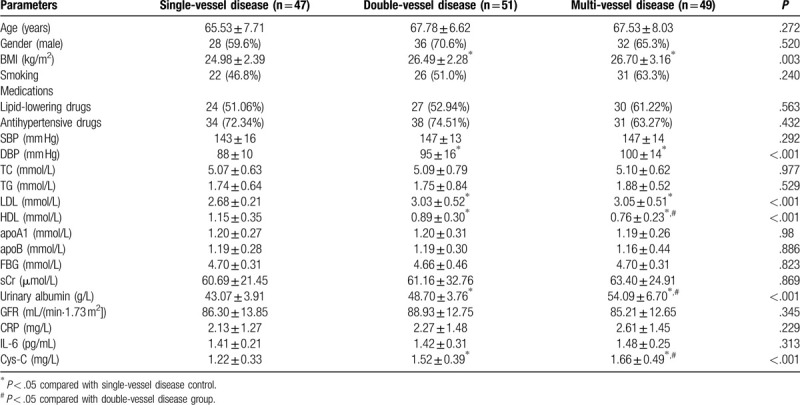
Clinical data of patients with hypertensive patients combined coronary heart disease with different lesion counts.

### 3.4 Correlation analysis between Cys-C and risk factors in patients with hypertensive patients with CHD

**Figure 4:**
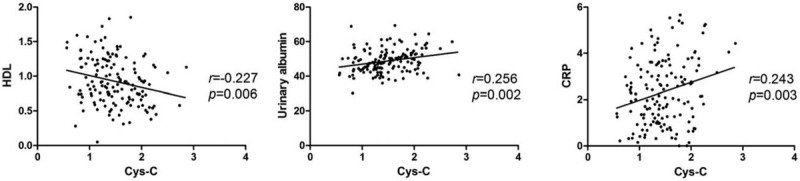
Correlations between Cys-C levels and HDL, urinary albumin and CRP in patients with hypertension and CHD. HDL (A) was significantly negatively correlated with Cys-C, and urinary albumin (B) and CRP (C) were significantly positively correlated with Cys-C.

To clarify the correlation between Cys-C and various clinical parameters in patients with EH + CHD, simple regression analyses were performed using Cys-C as a dependent variable (Table 3, Fig. 4).

**Table 3:**
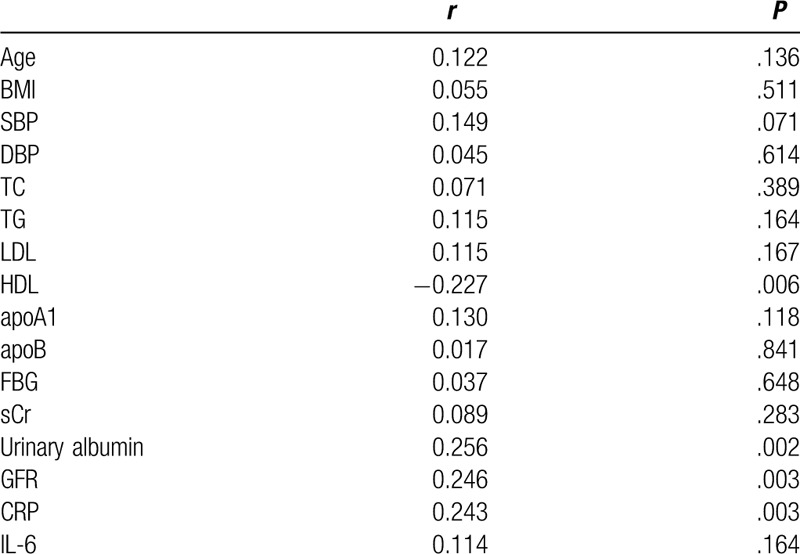
Correlations between Cys-C and various clinical parameters.

Urinary albumin, GFR and CRP correlated positively with Cys-C (urinary albumin: *r* = 0.256, *P* < .01; GFR: *r* = 0.246, *P* < .01; CRP: *r* = 0.243, *P* < .01), and HDL correlated negatively with Cys-C (*r* = −0.226, *P* < .01). However, no significant correlations were observed between Cys-C and age, BMI, DBP, SBP, LDL, TC, TG, apoA1, apoB, FBG, sCr, and IL-6.

### 3.5 Multivariate regression analysis of risk factors

To further analyze, the correlation between risk factors and hypertensive patients with CHD, we used CHD as a dependent variable, and age, BMI, DBP, SBP, LDL, HDL, apoB, urinary albumin, GFR, CRP, IL-6, and Cys-C was used as an independent variable for multivariate regression analysis. As shown in Table 4, age, LDL, apoB, IL-6, and Cys-C were independent risk factors for hypertensive patients with CHD, and Cys-C was the most harmful factor (OR = 84.953 [95%CI: 9.388–768.771], *P* < .05). Additionally, we found HDL was a protective factor for patients with hypertension and CHD (OR = 0.151 [95%CI: 0.023–1.009], *P* < .05). SBP, DBP, urinary albumin, CRP were not significantly related with hypertensive patients with CHD.

**Table 4:**
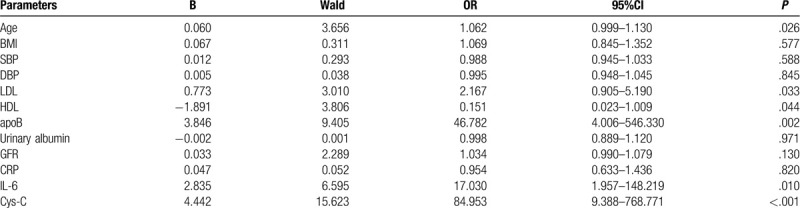
Logistic regression analysis of risk factors for coronary heart disease in patients with hypertension.

## 4 Discussion

Cys-C is a member of the cysteine protease inhibitor family and is considered to be the most critical inhibitor of cysteine proteases. Recent studies have shown that Cys-C is closely related to the occurrence and development of many cardiovascular diseases. Kestenbaum et al^[12]^ found that for every 0.2 mg/L increase in Cys-C concentration, the incidence of hypertension increased by 15%. Watanabe et al^[13]^ studied the relationship between Cys-C and target organ damage in patients with EH, and suggested that Cys-C may be a marker that can reflect the degree of target organ damage in patients with EH at an early stage. Early attention to the level of Cys-C in patients with hypertension is of guiding significance for the accurate assessment of the prognosis and treatment of hypertension. Additionally, Cys-C is closely related to heart failure, atherosclerosis, etc, and plays an important role in the pathophysiological changes of cardiovascular diseases.^[14]^ Disorders of cathepsin and Cys-C levels in the vessel wall may lead to the formation of atherosclerosis and affect its prognosis.^[15]^ Meanwhile, Cys-C can also be used as a risk factor for CHD and ischemic stroke, and predict carotid atherosclerosis, coronary stenosis and cardiovascular adverse events.^[5]^ Doganer et al^[16]^ found that with the increase of Cys-C level, the severity of coronary artery disease in patients with CHD increased significantly, and Cys-C level may reflect the degree of CHD. We found that the level of Cys-C was higher in the EH + CHD group than in the EH group and control group, respectively. And Cys-C was also associated with the severity of CHD. ROC analysis also suggested that Cys-C had a certain predictive value for the occurrence of hypertensive patients with CHD. Although Cys-C is considered to be a sensitive indicator for the assessment of GFR, the association between Cys-C and the development of coronary lesions is non-renal.^[17]^ Therefore, we believe that elevated levels of Cys-C are not only related to hypertension, but also closely related to the occurrence of CHD in hypertensive patients, and can be used as one of the factors predicting the severity of CHD and the severity of coronary artery disease.

We found that Cys-C was positively correlated with CHD risk factors such as CRP, urinary albumin and GFR in hypertensive patients, and negatively correlated with protective factor HDL. Under physiological conditions, Cys-C can prevent cells from being decomposed by endogenous and exogenous proteases by inhibiting the activity of cathepsins. When the vessel wall is damaged, the increased inflammatory mediator can cause a balance disorder between Cys-C and proteolytic enzymes in the vessel wall leading to destruction of arterial vascular integrity which ultimately leads to the formation of atherosclerosis.^[18]^ Simultaneously, the destruction of arterial integrity can induce elevated blood pressure,^[19]^ which eventually leads to coronary lesions.^[20]^ It has also been confirmed that Cys-C and its degradation products induce and aggravate atherosclerosis by interfering with the phagocytic and chemotactic functions of granulocytes involved in the inflammatory process.^[5]^ Balta et al^[21]^ showed that Cys-C can cause damage to vascular endothelial cells leading to a decrease in nitric oxide, which in turn causes platelets to adhere and aggregate, causing thrombosis and aggravating atherosclerosis. Furthermore, Wang et al^[22]^ found that under the protective effect of Cys-C, the decrease of vascular smooth muscle cell apoptosis can promote the progression of atherosclerosis caused by fibrous cap formation, which suggests that Cys-C is associated with abnormal lipid metabolism. Studies have confirmed that abnormal lipid metabolism is an independent risk factor for the onset of CHD, and is closely related to atherosclerosis.^[23]^ Therefore, we speculate that Cys-C may participate in the development of CHD by affecting the inflammatory response, extracellular matrix degradation, vascular wall remodeling, and lipid metabolism.

Cys-C, as an ideal endogenous marker reflecting GFR, is an early sensitive indicator of impaired renal function in normal serum creatinine, and the impaired renal function itself is a risk factor for CHD.^[24]^ Urinary albumin is an early sensitive indicator of renal insufficiency and is theoretically positively correlated with Cys-C,^[25]^ which was also confirmed in this study. Therefore, early impaired renal function or the early stage of impaired renal function may be associated with the development of CHD. In addition, high Cys-C is often associated with risk factors for CHD such as hypertension, hyperglycemia, hyperlipidemia, obesity, etc. Therefore, it is speculated that Cys-C affects the cardiovascular system through the combined effects of these factors.^[17]^ We also found that Cys-C is an independent risk factor for CHD in hypertensive patients. Therefore, Cys-C is expected to serve as a new screening indicator for better assessment of the risk of future cardiovascular events in hypertensive patients.

There are still many shortcomings in our research. First, CHD is caused by a variety of factors affecting different paths and factors affects and promotes each other, and the role of Cys-C in its pathogenesis needs to be further explored. The level of Cys-C in diabetic patients is significantly elevated, which can be used as an independent risk factor for predicting fasting blood glucose elevation.^[26]^ Therefore, we strictly excluded patients with diabetes and impaired glucose tolerance. Furthermore, the sample size in this study was small, so we cannot completely avoid the selection bias. Therefore, we still need further large-scale, multi-center, prospective studies to explore the relationship between Cys-C and hypertensive patients with CHD.

In summary, serum cystatin C levels in patients with hypertension are significantly elevated, and are closely related to the occurrence of CHD and the severity of CHD. In addition, serum Cys-C levels in patients with hypertension have predictive value for the occurrence of CHD and are one of the important risk factors for hypertensive patients with CHD.

## Author contributions

Miaohui Zhao and Tong Huang contributed the central idea, analysed most of the data, and wrote the initial draft of the paper. The remaining authors contributed to refining the ideas, carrying out additional analyses and finalizing this paper.
